# Supplementary material for: Transcriptome Analysis of Fibroblasts in Hypoxia-Induced Vascular Remodeling: Functional Roles of CD26/DPP4
Source: Int J Mol Sci. 2024 Nov 23;25(23):12599. doi: 10.3390/ijms252312599 (PMC11640941; doi:10.3390/ijms252312599)
Supplement: Supplementary file 1 [file ijms-25-12599-s001.zip › ijms-3280029-Supplementary_Figures_CD26_hypoxia.pdf]

Supplementary Figures

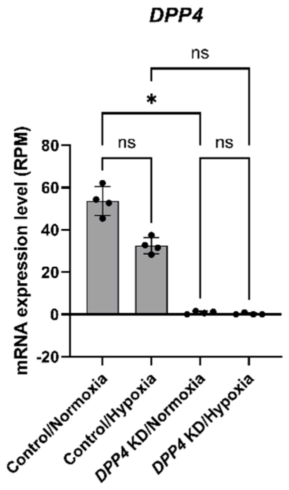

Supplementary Figure S1. The mRNA expression levels of *DPP4* measured by RNA sequencing of HLFs. The treatment of *DPP4* siRNA significantly downregulated the mRNA expression levels of *DPP4* in HLFs. * $p < 0.05$.

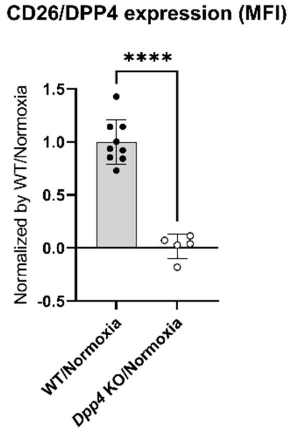

Supplementary Figure S2. CD26/DPP4 expression in whole lung cells of WT and *Dpp4* knockout (*Dpp4* KO) mice was measured using flow cytometry. CD26/DPP4 expression levels in whole lung were substantially lower or near zero in *Dpp4* KO mice. **** $p < 0.0001$.
